# American Academy of Dental Science

**Published:** 1896-07

**Authors:** 

**Affiliations:** American Academy Dental Science


### Reports of Society Meetings.

### AMERICAN ACADEMY OF DENTAL SCIENCE.

THE regular monthly meeting of the American Academy of Dental Science was held at Young's Hotel, Boston, Wednesday evening, February 5, 1896, at six o'clock, President Andrews in the chair.

*President Andrews.*—Gentlemen of the Academy: It becomes my pleasant duty to announce the programme of the evening, and I would state that we have with us as guests Edward Cowles, M.D., Superintendent McLean Insane Asylum; George H. M. Rowe, M.D., Superintendent Boston City Hospital; John L. Hildreth, M.D., Cambridge; Walter Channing, M.D., Brookline; Charles E. Woodbury, M.D., Massachusetts State Inspector of Alms and Reformatory Institutions, and Dr. Mackey, of Boston.

We have as our essayist to-night a gentleman who comes a long distance to give us, as he told me six months ago, what he himself considers one of his ablest papers, entitled "Dental and Facial Evidences of Constitutional Defect."

I have the honor to introduce to you this evening Eugene S. Talbot, M.D., D.D.S., of Chicago, Illinois.

(For Dr. Talbot's paper, see page 261.)

DISCUSSION.

*President Andrews.*—We have with us to-night gentlemen who are able to do this subject full justice, and it is with a great deal of pleasure that I call upon Dr. Edward Cowles to open the discussion.

*Dr. Cowles.*—I am very glad to have had the privilege of hearing Dr. Talbot's instructive and very interesting paper, and I regret very much that I am unable to contribute anything to the precise and scientific study which he has made of this subject. Those of us who as alienists are engaged in dealing with disordered mental manifestations do not often go into these matters in this way, although what I have heard to-night adds to my previous understanding of the matter of physical degeneration. And the statistics which have been given indicate its great importance to us in our study of mental diseases. I think the alienist who has made any extended study of mental diseases soon comes to recognize the fact that he may divide all his patients into two general classes. Very many of his patients who are insane may be said to be of the normal class. They are people of good heredity, who, under stress or wear of life, break down and become subjects of mental disorder, the result of physical and nervous exhaustion. We see this in cases of melancholia, and many other cases from which we expect a large proportion of recoveries. The other class, which we are latterly calling the "degenerates," are of that type of persons who have exhibited all their lives a certain degree of "nervous instability," as we say. These people are born with invalid brains; their nervous systems indicate an inheritance of weakness and conditions that are asthenic. Then there are some people who acquire that condition before coming to adult life, and in such cases the results which follow are about the same as in the cases of those who inherit it. The peculiar differences of the manifestations or symptoms in these cases are very striking, and it is of great interest to those of us who have studied these things to notice the peculiar variations from what might be called the regular forms of insanity that appear in these degenerates, or persons who tend to degeneracy in a greater or less degree. Now, if one can set up a comprehensive theory of this whole subject, which is very strongly suggested by Dr. Talbot's paper, it would certainly be very interesting, and I am inclined to think that there is a good deal in it.

In the cases of mental disorder which I first described among normal people, where we expect recovery after a due course of restorative treatment, securing the return of health by promoting nutrition and removing causes of strain, we are turning latterly, I think, to the theory of nutritional disorders and the means of repair; we are not looking so much for the causative influences as we must in those cases of heredity. We may regard such persons as being sound individuals, well endowed, but breaking down, as I said before, because of overwork, overstrain, they acquire conditions of fatigue and exhaustion, which means precisely disorders of the nutritional processes. These being corrected, the patient may recover; and such cases always present hopeful prognosis.

Turning to the other class, we see that the reason why they may yield sooner to influences which tend to unbalance the nervous and mental functions is that they are endowed with less than normal resistance to strain and stress. This is the same theory which we hold in relation to the physical side of life, the physiological or chemical theory,—that is, that impairment of function is due to disorders or derangements of the physiological or chemical processes of nutrition. These people are more susceptible and quicker yield to the influences that overbalance nervous energy.

It seems to me that Dr. Talbot has given a very distinct corroboration of the views I have had upon this subject, noting so plainly as he has the effects of trophic nutritional changes in their manifestations upon the conditions of the teeth and the bony structure of the organism. But when we come to study the more profound changes in the bony structure, such as he first noted, and attempt to assign a cause for the conditions that arise in the process of development that are due to congenital influences, I think we must have there a very rich field for study in finding conditions correlative with the mental changes, the observations of which make up the professional work of the alienist. I wish to thank him again for the very instructive paper, and to thank you, gentlemen, for your attention.

*President Andrews.*—There are many here present who will remember a delightful evening we had about two years ago, when a paper was read by Dr. Channing in a similar vein to the one we have heard to-night. I am sure we can consider ourselves very fortunate to have him with us again this evening to say a word on a subject in which we are mutually interested.

*Dr. Channing.*—It is a melancholy fact for us to believe, as stated by Dr. Talbot, that from fifty to seventy-five per cent. of your patients are degenerates. I think that is putting the percentage rather large. I remember at the Guiteau trial one quite brilliant expert alienist, who gave valuable testimony, made the statement that about one person in five was insane. When Judge Porter, the government counsel, got up to examine him, he looked quietly at the jury and said, "The witness has stated, gentlemen, that one person in every five is insane. There are twelve of you, and, according to his statement, two of your number must be insane." So it is easy enough to make these general percentages, but I feel that we have not yet got to a point where we can give the percentage of degenerates. In the first place, I think that word is used altogether loosely. I don't think we have any proper standard by which we can judge what a degenerate is. Among the authorities, it is understood that a person must present a certain number of stigmata to be classed as a degenerate, and that is the rule followed by Lombroso, whose investigations are of great interest, though he has based his statistics on a comparatively small number of persons. He has made a great number of measurements, and it is on the basis of these measurements, principally, that the statistics are founded from which his conclusions are derived, and we are now told that persons with certain sets of peculiarities are necessarily criminals or mentally defective. I must agree with David Nicholson, the superintendent of the Broadmoor Criminal Lunatic Asylum, in England, who takes issue not directly with Lombroso, but with the criminalists,—with the men who believe they can get at this thing from the anthropological point of view,—and thinks that they have failed to prove their case. On the same basis, Dr. Nicholson says we might establish a "doctorology," or a "parsonology," but how would it do to take a comparatively small number of clergymen and measure them very carefully, and, if they presented certain peculiarities, how would it do to conclude that all persons who presented about the same measurements and peculiarities must necessarily be clergymen? Suppose we should take a certain number of farmers and on investigation we find that they possess in common certain moral obliquities, and coincident with that we find that they present certain asymmetrical proportions, it would not follow that you could take all the farmers and classify them and make your deductions from these statistics. I do not believe that a few asymmetries necessarily prove that we have degenerates, and also do not believe that most of the asymmetries, upon which we are asked to believe certain statements, are as accurately determined as they should be. In the first place, every individual should not only be observed, but he should be carefully measured. Furthermore, we must not base our decision on the *bad points*, as Nicholson says, that we find in a man; we must also estimate the good points in him. If you are to decide that a man has certain mental or moral defects because by your measuring instruments he presents certain asymmetries, you naturally decide that it is a defect of evolution and there is nothing to be done for him, and yet the good qualities in that individual may perhaps, as a matter of fact, completely overshadow the defects. Therefore, I say that that is only part of the story. We have not yet arrived at the point where we know actually what a degenerate is; we have not had enough careful investigations to arrive at anything definite for a standard.

When I was with you before I referred to the measurements which I had taken of seven hundred of the insane. When I made these observations, several years ago, we did not estimate as carefully as we do now some of the physical stigmata. The measurements I took were of the head and outlines of the head, and therefore I should qualify a statement I made at that time in regard to no uncommon peculiarities presenting themselves as a rule in the upper class insane. I cannot say, as far as the palate was concerned, that I was struck by any peculiar difference showing itself between their mouths and the mouth of the average individual.

Going a step further, we cannot classify in a sweeping way as degenerate persons who present even mental defects, because, as Dr. Cowles has told you, some of those who have acquired mental disease may present peculiarities similar to those of the congenital class, but it is using a very strong word to call them "degenerates." They are individuals who present phases of evolution, disorganization, and thus include all various forms of mental weakness and defect and peculiarity and originality.

As to idiots: Idiots certainly should present very marked degenerate physical features, and they naturally do, because they are the lowest stage of degenerates. They represent varying degrees of arrest of development, and you cannot help in such cases finding stigmata of various kinds and sorts; but I am certain that even in idiots we overstate this question of physical degeneration. I cannot speak as positively as you can about the teeth, not having had such extensive opportunities for observation, and perhaps a more critical observer could notice defects that I would not. I hope Dr. Talbot will come and see my casts, and perhaps he can elucidate something from them. I have always held that the only proper way to get the asymmetry of the palate is to take a cast of it. Of course the reproduction is not perfect, but there you have something on which you can study, and I think it sufficiently accurate for our purpose. This plan was not followed by Dr. Clouston in his study of the mouths of six hundred persons from which he has made up the percentages which he has presented to us. The persons examined included criminals, insane, idiots, boys in a school, epileptics, etc., and to show you how these examinations were made, he states that over two hundred convicts in a prison were examined in two mornings. Well, upon such a basis as that, it seems to me his observations are of somewhat questionable value. He divided up his palates into three classes,—*typical*, *neurotic*, and *deformed*,—and he got of the typical kind eleven per cent.; neurotic, twenty-eight per cent.; deformed, sixty-one per cent. This tremendous percentage of sixty-one per cent. was arrived at in the way that I have detailed. I have one thousand good casts of the palates of idiots, and I will give you the percentages that I got from a careful study of these casts. I did not feel that I really knew what a typical mouth was, so I did not use that term, but put them under the classification of "average," and I found that fifty-two per cent. of them were average, 37.2 per cent. were neurotic, and 10.8 per cent. were deformed. Mr. Edward Atkinson, who, we all know, is a man fond of statistics, has made a revision of the old phrase, "figures lie," and puts it a little differently; he says, "liars figure." It is very evident that the figures have lied in either one case or the other; but I feel secure in my position, because I have the casts to back me. I cannot agree with Dr. Clouston in these statistics, though he is one of the best authorities on mental diseases that we have.

In speaking of the condition of the teeth that Dr. Talbot finds among the insane, I do not think in my experience I have found that the teeth of the insane are in very much worse condition than might possibly be expected from the care which their teeth naturally receive. You must remember that these people take no care of their teeth, and taking into consideration also the kind of food that they have in the public hospitals, what can you expect under such conditions? Idiots also neglect their teeth; but, in spite of all this, I have been surprised to find how good they were. To refer again to the statistics I had of these thousand casts of idiots I have put down 93.2 per cent. of their teeth as being average (that refers to the size), 1.2 per cent. large, 5.6 per cent. small, 70.5 per cent. regular, 29.5 per cent. irregular, forty-five per cent. decayed, and in eleven per cent. dentition was delayed.

There is just one point more on which I will speak in regard to the insane. If we can rely upon the English statistics of lunacy, the increase is not very rapid,—that is, the asylums have ceased to fill up as they did in former years. Of course, the asylums are always full; but, taking all the cases which are registered in England, it is stated that the number of cases of mental disease is not increasing very rapidly, and those who enter the insane hospitals are more often than formerly from the class Dr. Cowles and I have both spoken of,—of the constitutional alienations of the degenerate class. We get fewer of the acute cases of acute mania and more of these cases, which, as I said before, are the result of constitutional defects.

*President Andrews.*—The Academy had invited to be present here to-night and take part in the discussion, Dr. Fernald, of the Home of the Feeble-Minded, and up to ten or fifteen minutes before our dinner he was expected to be here, but at that time I received a note from him, stating that he was unable to be present, which he regretted very much, as he wished to be here and meet Dr. Talbot. Fortunately, we have with us a gentleman who has given this subject a good deal of attention, and it is with great pleasure that I introduce to you Dr. Chas. E. Woodbury.

*Dr. Woodbury.*—I think your President has a little overstated the matter when he says that I have given much attention to this thing, because, unfortunately for me, I have not. Of course, a person being connected with institutions mostly caring for the insane for twenty years could not help noticing some of these points, but I wish to thank Dr. Talbot for the information I have received from his paper. In my position as State inspector of institutions for the State Board of Lunacy and Charity, a large number, practically all the insane of the State, pass under my eye, and on this account I wish that I might add something to this discussion, but I certainly must express to you my pleasure at being here, and great gratification at hearing this very interesting paper. It will serve as a stimulus to me, as I go about in the different asylums, to observe these appearances which he has so carefully noted in his paper.

I may say to you that we have about seven thousand—a little less—in our insane asylums and in the almshouses in this State, which is quite a small percentage, the population being about two million five hundred thousand. Of this number a little over five thousand are in the State institutions proper. We have some seven or eight hundred in the town and city almshouses; that includes part of what we might not properly call insane, but feebleminded, and, as I said, going through this large army of degenerates,—if we can call them so, although I have never regarded them as such, as has been stated before here this evening,—I have had many opportunities of noting physical defects, yet the paper of Dr. Talbot will help me very much in the study of these unfortunate beings that are confided to our care.

*Dr. Brackett.*—It seems to me that there is a great deal that might be said in this connection, yet I hesitate to begin to speak, fearing I may say too much, and knowing that others here are much more capable of discussing the subject than myself. The paper presents so many phases that the possibilities for discussion seem to be almost endless.

I think dentists in recent years, more than formerly, have come to regard many of the local lesions that come specially within our province as being dependent upon systemic conditions rather than local causes. In multitudes of ways we appreciate that fact. The connection between gouty diathesis and erosions and abrasions of the teeth, between rheumatic affections and diseases of the kidneys and deposits of tartar in the mouth and other relations are becoming more and more noted, so that we now look for poor nutrition, poor innervation, and all of that in connection with the affections which we especially treat. For instance, there came under my observation a young lady whose teeth were all in a good condition of repair at a particular date, but she had a severe illness, the exciting cause of which was prolonged sea-bathing,—remaining in the water until she was chilled, followed by an illness of more than a year, and the development in eighteen months of thirty-six new cavities. In multitudes of ways, too numerous even to think of making allusion to in the short time we have for discussion, we see the connection between systemic condition and local expression. I have a patient whose mouth I first examined some twenty-two years ago, and she has remained continuously in my charge ever since. When I first saw her teeth I thought that they were likely to need very little repair during the rest of her life, but she began to have a series of troubles, family bereavements, a changed home, anxiety over money matters, general worry, and all that sort of thing, and for a score of years her teeth have needed a great deal of attention. There have been successive deaths of pulps and consequent treatment, and I do not think that for several years past there have been three successive months during which she has not been in my chair. During a part of this time her nervous condition was such that there were quite marked indications of locomotor ataxia and various other neurotic expressions. I mention this as one of the multitudes of instances where I have noted this coincidence of tooth-failure and nervous troubles. I think we are appreciating more than ever before how innervation and nutrition are the foundations upon which rest the mental and physical well-being of the individual, and that the lack of general well-being is one of the many circumstances which affect the character of the teeth.

With reference to hereditary influences and tendency to degeneration, numerous instances passed through my mind as I was listening to the paper, especially in cases of multiple birth, which seemed in a way to corroborate, at least partially, some of the doctrines. In one family in which in different members there have been expressions of epilepsy, tuberculosis, and rachitis, I have found several instances of what has been denominated spontaneous death of the pulps. In the case of one daughter there has been not less than three sound upper molars with dead pulps.

I am a great believer in the doctrine of maternal impression,—of the influence of the mother's system and mother's mental condition during pregnancy upon the well-being of the child. I have an instance in mind which I think is a good illustration, and the facts are quite within the knowledge of the gentleman on my left. There were three daughters in the family. The older daughter, it is right to say, was of feeble mind; the second possessed good general intelligence and capacity, and was free from any marked neurosis. She was born, as I have heard her mother say, under more favorable conditions, the mind of the mother having been in a more tranquil state and the general health good. During the months previous to the birth of the third daughter the mother was in a condition of great anxiety and worry, even fear, on account of a number of circumstances by which she was surrounded. The difference between the second and third daughter was very noticeable, the youngest being noted for her extreme timidity, which was apparent in her walk and in her manner of speaking. She was extremely nervous, any little noise coming unexpectedly would give her a start, and she would faint away on very slight provocation. Soon after she passed out of her teens she became an inmate of the State Insane Asylum, and shortly afterwards died. I think if we try to bring to our minds circumstances within our knowledge of people whom we have known, we shall find ample evidence to corroborate the doctrines of the paper with reference to the inevitableness of the expression of hereditary traits of various kinds, whether it be the murderous disposition or kleptomania, dipsomania, prostitution, or any of these abnormally-debased expressions of humanity. I noted in a newspaper, while I was on the train coming here to-night, an editorial commenting on an article in a recent number of the *Cosmopolitan Magazine* by Margaret Deland. The story or sketch runs something like this:

A certain woman devoted herself to the work of reclaiming a particular prostitute. She gave the matter earnest attention, protecting, encouraging, and shielding the woman in every way for a long period, and while under this protection the reformation seemed to be sincere; but, just as soon as that was withdrawn, the prostitute recurred to her former ways and soon came to the end of her life. The suggestion of the sketch is that such persons are mental degenerates, and the newspaper in its criticism states that work for their reclamation is effort not well directed. Certainly there comes out of this the moderate deductions that those people who in their mental make up exhibit no marked prominence of any kind of insanity ought to be particularly thankful; that many of our mental and moral degenerates act from impulses that are entirely beyond their control and for which they cannot be held responsible; and that the only help we can give them is to be charitable with them and deal with them considerately, as I believe is done by those who are in charge of the almshouses, the insane asylums, and the reformatory institutions generally of our New England States.

*Dr. J. L. Williams.*—I can only express my gratification at the systematic thoroughness, and patient, persistent investigation shown in Dr. Talbot's paper. It clearly presents the fact that a "truism is defective in not recognizing any but an immediate cause."

The *immediate* cause, whether chemical or bacterial, does not *fully* explain dental caries in constitutional diseases, or in overstrain of vital powers from various causes, the bony system, as he says, being most completely under the control of the nervous system. As Dr. S. B. Palmer has said, "There are deeper causes than chemical or bacterial ones." This subject of vitality is the root of the science of biology, of which our specialty, with general medicine, is but a branch, and the more we know of the root the better and sounder knowledge we shall have of the branches.

My own general observations of the connection of morbid states of the teeth and mouth, with conditions of nervous overstrain from constitutional effects, or from too continuous testing of the vital powers in various ways, led me, several years ago, to write a short paper on the subject, which I read before this Academy in November, 1872, and which was printed in the *Dental Cosmos* in December of that year. It was entitled, "The Influence of the Nervous System on the Health of the Teeth and Mouth." I read another paper of similar bearing in our section of the American Medical Association, at Washington, on May 8, 1884. Its title was "Overdraft of Vital Power as affecting General and Special Health."

It is refreshing to see the conclusions from merely general observation confirmed by the laborious statistical and scientific work of men who have patience to do it. So was also the case with the plan of preparatory treatment of delicate teeth, founded on general observations (as the farmer or fisherman would make a diagnosis and prognosis of the weather), but which, by the labors of a Miller, a Black, an Andrews, and others, have been found to have scientific basis quite as exact as that of the weather bureau.

But this deeper and fundamental principle of vitality or nerve power, Dr. Talbot seems to have especially delighted in elucidating by his patient work of statistical observation.

*President Andrews.*—I recall a pleasant visit to the home of our essayist in Chicago some years ago. I remember with how much interest he took me to see some degenerates, called the Aztec children, museum freaks, for an examination, and I remember how critically he examined them. I believe he has already examined the murderer Holmes, and has the cast of his mouth. He has also made a careful examination of the murderer Gilbert, and has the cast of his jaws, and is trying to get a chance to see Pomeroy, the murderer. Indeed, he seems to be a busy man, so busy he could not accept our hospitalities. It is by this constant work that he is able to present to us such extensive investigations as he has tonight.

*Dr. Fillebrown.*—The subject has been so thoroughly discussed by those who have spoken before me that there is not much that I can say, but I wish to put myself on record as being interested in this paper. I have read the articles which have been published in various magazines with interest. And after hearing the paper tonight I am very much strengthened in the conclusions I had before.

The author's propositions are, first, that we are the result of our environment, and he refers to the children of the siege of Paris for example. This shows plainly that a person may be born of a perfectly normal germ, from a healthy male parent impregnating a healthy ovum from a healthy female parent, and yet during development of that germ circumstances may arise which may cause it to be improperly developed. Now, that will undoubtedly produce a degenerate offspring. It is, of course, the degeneracy of accident and not of constitution nor of heredity, and it seems to me that more than fifty per cent. of our physical failures are the result of such accidental circumstances, and consequently it is rather difficult to judge to what extent heredity is responsible for physical defects.

This question of environment extends still further, and brings us to another conclusion of the paper, which answers itself,—that growth and conditions are subject to the accidents of subsequent life. Dr. Cowles and Dr. Channing both spoke of the power of our resistance. Human nature can do just about so much and no more; when that power is exhausted then we go down. This point of the limit of resistance of the mental powers is reached at different periods of life. It may happen in infancy, youth, middle life, or in old age,—it depends entirely on the surrounding circumstances. A great many persons will live to a good old age and die a natural, easy death, and be considered perfectly normal, who, had they been put under different circumstances, would have been unable to withstand the mental strain put upon them, and would have gone into the asylum. It is so much a question of how much resistance we have, it is difficult to tell what is degenerate or otherwise. If you take a truck-horse and speed him with a trotting horse he will at once appear to be degenerate, and, *per contra*, if you put the trotting horse against the dray-horse when it is a test of strength and endurance, of course the trotting horse will break down; put them both under the circumstances in which they ought to live, they are both normal instead of degenerate. This same question of adaptability to environment and the results of outside circumstances should be taken into consideration before classing a person as a degenerate. At the latter part of the paper the essayist speaks of the results of these circumstances, and refers to the observations in his own practice. He has noticed patients who go on in life, and so long as their resistance lasted they were normal; there was nothing to indicate that they were not equal to the average person in mental and physical health, but as soon as their resistance failed they began to exhibit these mental and physical defects, and the characteristics of degeneracy became evident.

To understand pathology, we have to begin first with the study of physiology in order to be able to distinguish between health and disease. We must know, first, what is the normal condition, the normal action, before we can declare what is abnormal or imperfect. It strikes me that the plan followed by Dr. Talbot in making up his statistics, either by observation or measurements, fails a little in not showing us what we should expect in a normal individual. We meet with many cases of persons in first class physical and mental health who do not present normal mouths. Suppose we should go over to Harvard College and make examinations of the mouths of the three thousand students there. We should expect to find there a high average of both mental and physical development, but how much will the percentages differ from the insane or others? I imagine very little; I think you will find about the same variation. There is no such thing as a typical mouth, because there are wide variations within normal limits, and we are not able to decide what are the extremes.

I would like to ask the essayist whether, in making up his statistics, he has completely analyzed them so as to understand whether the patient under examination should be classed under the head of acquired degeneracy or constitutional degeneracy. For instance, if a deaf and dumb person is under examination, has he discriminated between the two classes, the congenital and the acquired, and, if a case of mental disease, has he recognized the two classes that Dr. Cowles so clearly and forcibly defined here to-night?

*President Andrews.*—A word in regard to the formation of the tooth. The full size and width of the point is formed in embryonic connective tissue. This is covered by its enamel organ. These tissues calcify and growth goes on normally until scarlet fever, measles, or some other disease occurs. During this illness there is an arrest of growth, the tissue forms faulty or shrunken. When health returns the growth goes on normally again, and the tooth is bound to attain to its full size, as Dr. Talbot says.

If there is nothing more to be said, Dr. Talbot has the floor to finish the debate if he so desires.

*Dr. Talbot.*—I have a very little to say in rebuttal of the remarks that have been made this evening. Dr. Channing has taken the opposite side of my paper to a certain extent, and makes the point that he is very sorry if it be true that so many of our patients are degenerate. I regret that he was obliged to leave us to catch his car.

Dr. Channing is looking into the future; we cannot go beyond what the present offers in regard to the study of degeneracy. We must take the accepted views that are held by alienists and neurologists at the present day. In France, Austria, and Italy, where most of this work has been done, the subject has been earnestly studied for the past fifteen or twenty years, and naturally more advance has been made in other countries; they claim that one deformity means nothing; that an individual must present at least three or four deformities of different organs of the body or parts of the face. That is the rule we must follow in our investigations to-day, and what the rule may be twenty-five or fifty years in the future. I agree with Dr. Channing that the term degenerate is rather indefinite, but we must take the rule as laid down at the present day by those who have given so much more time and study. If you will study your patients as I have, you will come to the same conclusion. I am not speaking at random; I am speaking from facts.

Dr. Channing also criticised the statistics given by Dr. Clouston with regard to the deformities of the mouths of idiots. It might be well to state that Dr. Clouston is one of the ablest investigators in the world; I think he will rank as one of the best men who has ever written on the subject of mental diseases. He has written a number of works, but his work upon "The Neuroses of Development" is one of the best that I have read touching upon this subject of the palate. I am sorry to say, however, that he is not as well posted as we dentists in regard to the jaws, and in making up his statistics he puts down under separate headings in his classification the neurotic jaw and the degenerate jaw. Now, if you ask a neurologist to tell you the difference between a neurotic individual and a degenerate, he will say to you that a neurotic is unstable through the influence of a weakened nervous system, and that a degenerate is a little more unstable; at best it is a question only of degree. Therefore Dr. Clouston makes the fatal error in his classification by classifying the same condition, as we understand it, under the separate heads of neurotics and degenerates.

Dr. Channing also speaks of the difference in the sizes of the teeth; that he has found the size of the teeth of the degenerate to be equal to that of the average healthy individual. We do not expect to find any difference in the sizes of the teeth. If the teeth do not vary in size to-day more than they did three thousand years ago, why do they not? It is very easy to understand. In our histological studies we learned that the teeth attain to their typal limit by the sixth week, and if they do vary in size that change must take place before six weeks. After a tooth has attained its size and form no condition of the body can change that size, because it has got its growth before that period. The foundation for the development of the teeth is completed by the eighth or ninth month, and as long as the patient lives no change can take place; therefore the child does not inherit nor do trophic changes in after life affect the structure of the teeth to the extent of producing a degenerate tendency in either the form or the size; consequently in degenerates we would not expect to find a great difference in condition of the teeth.

In the course of Dr. Fillebrown's remarks he asked if I made any distinction between the acquired degeneracy and the constitutional. Certainly I do. I will confess that in my first work I did not make this distinction, because the subject was entirely new to me, and I hardly knew what facts had to be considered, but I distinctly understood the difference between inherited conditions and those of trophic changes, and my statistics are made up accordingly.

I agree with Dr. Brackett in regard to the influence of heredity in its bearing on this subject.

*Dr. Clapp.*—I wish to offer a motion for a vote of thanks to Dr. Talbot for giving us this very able paper. All of us are busy trying to stop the ravages of decay, and when any man can come before us as busy as we are, but who has more force perhaps, and who has spent so much time as Dr. Talbot has investigating and tabulating such matter as he has brought before us tonight, the least we can do is to express our appreciation of it, so I have great pleasure in offering this motion for a vote of thanks.

Unanimously carried.

William H. Potter, D.M.D.,
*Editor American Academy Dental Science.*

